# Understanding Secondary Sarcopenia Development in Young Adults Using Pig Model with Chronic Pancreatitis

**DOI:** 10.3390/ijms25168735

**Published:** 2024-08-10

**Authors:** Ewa Tomaszewska, Dorota Wojtysiak, Agnieszka Grzegorzewska, Małgorzata Świątkiewicz, Janine Donaldson, Marcin B. Arciszewski, Sławomir Dresler, Iwona Puzio, Sylwia Szymańczyk, Piotr Dobrowolski, Joanna Bonior, Maria Mielnik-Błaszczak, Damian Kuc, Siemowit Muszyński

**Affiliations:** 1 Department of Animal Physiology, Faculty of Veterinary Medicine, University of Life Sciences in Lublin, 20-950 Lublin, Poland; iwona.puzio@up.lublin.pl (I.P.); sylwia.szymanczyk@up.lublin.pl (S.S.); 2 Department of Animal Genetics, Breeding and Ethology, Faculty of Animal Sciences, University of Agriculture in Kraków, 30-059 Kraków, Poland; dorota.wojtysiak@urk.edu.pl; 3 Department of Animal Physiology and Endocrinology, University of Agriculture in Kraków, 30-059 Kraków, Poland; agnieszka.grzegorzewska@urk.edu.pl; 4 Department of Animal Nutrition and Feed Science, National Research Institute of Animal Production, 32-083 Balice, Poland; malgorzata.swiatkiewicz@iz.edu.pl; 5 School of Physiology, Faculty of Health Sciences, University of the Witwatersrand, Parktown, Johannesburg 2193, South Africa; janine.donaldson@wits.ac.za; 6 Department of Animal Anatomy and Histology, University of Life Sciences in Lublin, 20-950 Lublin, Poland; mb.arciszewski@wp.pl; 7 Department of Analytical Chemistry, Medical University of Lublin, 20-093 Lublin, Poland; slawomir.dresler@umlub.pl; 8 Department of Plant Physiology and Biophysics, Faculty of Biology and Biotechnology, Maria Curie-Skłodowska University, 20-033 Lublin, Poland; 9 Department of Functional Anatomy and Cytobiology, Faculty of Biology and Biotechnology, Maria Curie-Sklodowska University, 20-033 Lublin, Poland; piotr.dobrowolski@umcs.lublin.pl; 10 Department of Medical Physiology, Chair of Biomedical Sciences, Institute of Physiotherapy, Faculty of Health Sciences, Jagiellonian University Medical College, 31-501 Kraków, Poland; joanna.bonior@uj.edu.pl; 11 Chair and Department of Developmental Dentistry, Medical University of Lublin, 20-081 Lublin, Poland; mielnikmb@gmail.com (M.M.-B.); damiankuc@vp.pl (D.K.); 12 Department of Biophysics, Faculty of Environmental Biology, University of Life Sciences in Lublin, 20-950 Lublin, Poland; siemowit.muszynski@up.lublin.pl

**Keywords:** pig, chronic pancreatitis, sarcopenia, muscle fibers

## Abstract

Chronic pancreatitis (CP) in young individuals may lead to disease-related secondary sarcopenia (SSARC), characterized by muscle loss and systemic inflammation. In this study, CP was induced in young pigs, and serum levels of key hormones, muscle fiber diameters in various muscles, and the mRNA expression of genes related to oxidative stress and programmed cell death were assessed. A decrease in muscle fiber diameters was observed in SSARC pigs, particularly in the longissimus and diaphragm muscles. Hormonal analysis revealed alterations in dehydroepiandrosterone, testosterone, oxytocin, myostatin, and cortisol levels, indicating a distinct hormonal response in SSARC pigs compared to controls. Oxytocin levels in SSARC pigs were significantly lower and myostatin levels higher. Additionally, changes in the expression of catalase (*CAT*), caspase 8 (CASP8), B-cell lymphoma 2 (*BCL2*), and BCL2-associated X protein (*BAX*) mRNA suggested a downregulation of oxidative stress response and apoptosis regulation. A reduced *BAX*/*BCL2* ratio in SSARC pigs implied potential caspase-independent cell death pathways. The findings highlight the complex interplay between hormonal changes and muscle degradation in SSARC, underscoring the need for further research into the apoptotic and inflammatory pathways involved in muscle changes due to chronic organ inflammation in young individuals.

## 1. Introduction

Sarcopenia, according to the definition of the International Working Group on Sarcopenia (IWGS) in 2009, is a prevalent condition characterized by a progressive loss of muscle mass and strength that typically begins after the age of 30 and accelerates after 50 years [1]. Although the term has been increasingly recognized in clinical settings, a universally accepted definition has been elusive. Subsequent consensus definitions provided by the IWGS, the European Society of Clinical Nutrition and Metabolism Special Interest Group (ESPEN-SIG), the European Working Group on Sarcopenia in Older People (EWGSOP), and the Society of Sarcopenia, Cachexia and Wasting Disorders (SSCWD) have contributed to the evolving understanding of sarcopenia [2]. In 2018, sarcopenia was further refined with the recognition of muscles as secondary endocrine organs [3]. Despite these advancements, a global consensus on the definition remains incomplete, contributing to the underdiagnosis of sarcopenia in clinical practice.

Current diagnostic approaches for sarcopenia often rely on advanced imaging techniques, such as dual-energy X-ray absorptiometry (DEXA), computed tomography (CT), magnetic resonance imaging (MRI), and clinical ultrasound [4]. Among these, MRI and CT are considered the gold standards, providing detailed and accurate measurements of muscle mass, density, and fatty infiltration [5]. Currently, the most reliable basis for detecting sarcopenia is not only confirming low muscle mass with a reduced amount and altered quality of muscle fibers, but also confirming low muscle strength, muscle performance, and physical fitness. For this reason, bioelectrical impedance analysis (BIA) is largely utilized for the assessment of skeletal muscle mass [6]. However, there is a lack of consensus on or standardization of different measurement methods and diagnostic radiological cutoff points [3]. Diagnosing sarcopenia requires reliable quantification of muscle mass using valid, repeatable, and cost-effective tools. Although many tools are available, the lack of population homogeneity and diverse research conditions complicate the systematic implementation of these tools and accurate diagnostics [7]. In sarcopenia, muscle quality is also altered due to disruptions in the micro- and macroscopic aspects of muscle architecture and composition. Thus, proper investigation of muscle cells is available through muscle biopsy, which, along with typing muscle fibers, is an excellent tool for evaluating muscle quality [7].

Because changes in skeletal muscles are largely attributed to the complex interactions among factors that include alterations of the neuromuscular junction, endocrine system, growth factors, and muscle protein turnover, as well as behavior-related and disease-related factors, the identification of SARC is complicated; and the identification of a single biomarker of SARC is unreliable, due to its “multifactorial” pathogenesis involving a multitude of pathways. SARC development is multifactorial, involving intricate interactions between programmed cell death mechanisms and oxidative stress. These processes lead to muscle fiber atrophy, impaired regeneration, and infiltration of non-muscle tissues, collectively contributing to the progressive loss of muscle mass and function [8]. SARC development involves both programmed cell death mechanisms and oxidative stress. Apoptosis increases in aging muscles, leading to muscle fiber loss, while autophagy dysregulation results in either excessive or insufficient degradation of cellular components, both of which contribute to muscle degradation. Necroptosis, another form of cell death, triggers inflammation and worsens muscle loss [9]. Oxidative stress, characterized by an imbalance of reactive oxygen species (ROS), damages muscle cells, with mitochondrial dysfunction exacerbating ROS production and reducing ATP, further weakening muscles [10]. Chronic inflammation, driven by oxidative stress, accelerates muscle breakdown and has a direct effect on muscle protein turnover [11]. Together, these mechanisms cause muscle fiber atrophy, impair muscle regeneration by affecting satellite cells, and lead to the infiltration of connective and adipose tissues, disrupting muscle structure and function.

New evidence indicates that sarcopenia is also a concern among obese and chronically ill patients, suggesting that the actual number of individuals at risk for adverse outcomes is significantly higher than previously thought, with personal, social, and economic costs exceeding expectations [7,12].

Sarcopenia is classified into primary, age-related sarcopenia (PSARC), and secondary, disease-related sarcopenia (SSARC) [13,14]. SSARC has multifactorial origins, including age-dependent biological changes, lifestyle habits, disease-causing factors, and disease-dependent biological changes, such as neurological disorders, dementia, Alzheimer’s disease, lung diseases, diabetes mellitus, cancer, organ failure, inflammation, immobilization, and obesity [15]. Disease-related SSARC is observed in patients undergoing surgical procedures (e.g., cardiac, orthopedic, gynecological, or vascular surgeries) or in those with gastrointestinal cancers (e.g., liver, colon, stomach, or pancreas) and other chronic illnesses, such as inflammatory bowel disease or pancreatitis [16,17,18,19,20,21,22,23,24,25,26]. While age-related PSARC is relatively well understood, disease-related SSARC, especially when multiple factors beyond aging are involved, remains less defined [13]. SSARC can result from a complex interplay of etiological factors, including cancer, organ failure, inflammation, immobilization, and obesity, and is considered to be more prevalent than primary age-related sarcopenia, even affecting younger individuals [27,28,29,30,31,32]. Chronic pancreatitis (CP), a progressive inflammatory disease, is a well-known contributor to systemic inflammation and rapid muscle degradation. CP is associated with increased intestinal permeability, which can impair nutrient absorption and lead to overall systemic weakness. The chronic inflammatory state induced by CP, combined with alterations in pancreatic function and potential malnutrition, exacerbates the risk of developing SSARC. Over time, chronic inflammation and cancer can further exacerbate SSARC [33,34].

Thus, a novel animal model of CP has been proposed [35,36]. This model is particularly relevant because it reflects the complex interplay of chronic disease in a controlled setting and provides insights into how mid-grade systemic inflammation develops in young individuals.

By applying this model, it is hypothesized that experimental CP in young animals linked with body mass loss can result in SSARC over time. For this purpose, cerulein-induced CP was triggered in young pigs to advance the understanding of how chronic disease affects muscle in young individuals.

Therefore, to investigate sarcopenic changes, skeletal muscles with distinct functions—the dynamic musculus triceps brachii (MT) and the static musculus longissimus lumborum (ML), along with the diaphragm and heart—were selected. Assessments were made of the number and percentage of muscle fibers of types I, IIa, and IIb, and measurements of their diameters were performed to evaluate structural changes. In addition, blood serum levels of myostatin, a negative regulator of muscle growth, and oxytocin, which has anti-inflammatory properties and inhibits myostatin, were analyzed. To explore the effects of chronic stress and inflammation on the hypothalamic–pituitary–adrenal (HPA) axis, the DHEA/cortisol ratio was assessed, and testosterone levels were measured as an indicator of anabolic activity. Finally, to examine mechanisms related to oxidative stress and programmed cell death in muscles caused by mid-grade chronic inflammation, the expressions of genes related to oxidative stress (*SOD1*, *CAT*) and apoptosis (*CASP3*, *CASP8*, *BCL2*, *BAX*) were quantified, and the *BAX/BCL2* mRNA ratio was calculated as an indicator of apoptosis.

## 2. Results

### 2.1. Muscle Fibers

**Figure 1:**
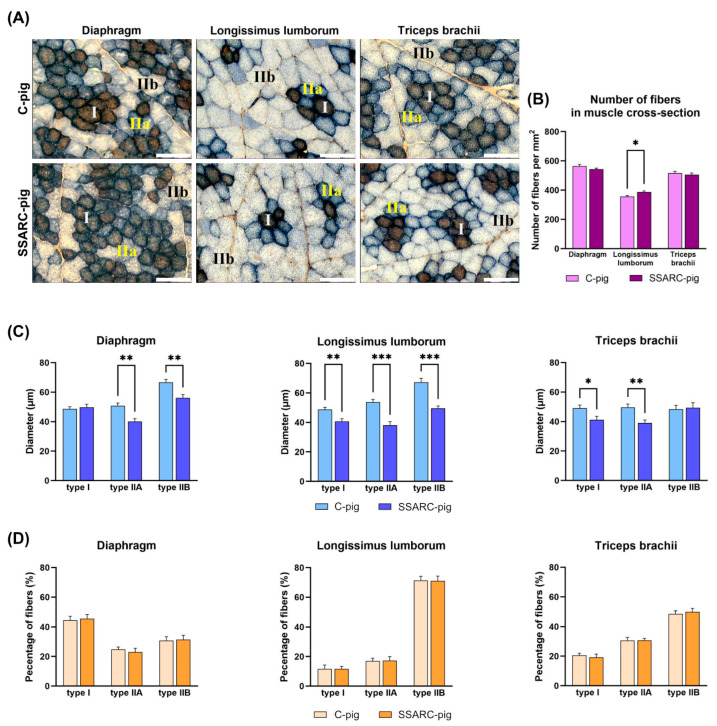
Muscle histomorphometry. (**A**) Representative images of the NADH-TR and MyHC stained sections of the diaphragm, the longissimus lumborum muscle, and the triceps brachii muscle in the control (C-pig) and cerulein-injected (SSARC-pig) groups, showing muscle fiber type variability. Type I fibers are stained brown, type IIa fibers are stained blue, and type IIb fibers are stained white; (**B**) Comparison of numbers of muscle fibers in the cross-section of the diaphragm, the longissimus lumborum muscle, and the triceps brachii muscle; (**C**) Comparison of the diameter of type I, IIa, and IIb muscle fibers in the diaphragm, longissimus lumborum muscle, and the triceps brachii muscle; (**D**) Comparison of the percentages of type I, IIa, and IIb muscle fibers in the diaphragm, longissimus lumborum muscle, and the triceps brachii muscle. In graphs, data are represented as the mean ± SE values (n = 5 in each group). Statistical significance: * *p* < 0.05, ** *p* < 0.01, *** *p* < 0.001 (two-tailed *t*-test adjusted for multiple comparisons using the Bonferroni method). In images in (**A**), all the scale bars show 100 μm.

Figure 1A shows representative images of muscle fiber type variability in the diaphragm, the LM muscle, and the MT muscle in the control and SSARC groups. While the overall number of muscle fibers in the muscle cross-sectional area increased only in the LM muscle (Control: 356 fibers/mm^2^, SSARC: 387 fibers/mm^2^; Figure 1B), sarcopenic changes were evident in all analyzed muscle types, as indicated by alterations in the diameters of different muscle fiber types (Figure 1C). In the diaphragm, the diameter of type I and IIa muscle fibers decreased in the SSARC group compared to the control group (type I: Control: 50.8 µm, SSARC: 40.1 µm; type IIa: Control: 66.7 µm, SSARC: 56.1 µm). In the LM muscle, the diameters of all muscle fiber types decreased (type I: Control: 48.8 µm, SSARC: 40.6 µm; type IIa: Control: 53.8 µm, SSARC: 38.2 µm; type IIb: Control: 67.2 µm, SSARC: 49.6 µm). In the MT muscle, a decrease in fiber diameter was observed in the SSARC group for type I and type IIa muscle fibers (type I: Control: 49.1 µm, SSARC: 41.2 µm; type IIa: Control: 49.8 µm, SSARC: 39.1 µm). However, the percentages of the various fiber types did not change (Figure 1D).

### 2.2. Sarcopenic Indicators

**Figure 2:**
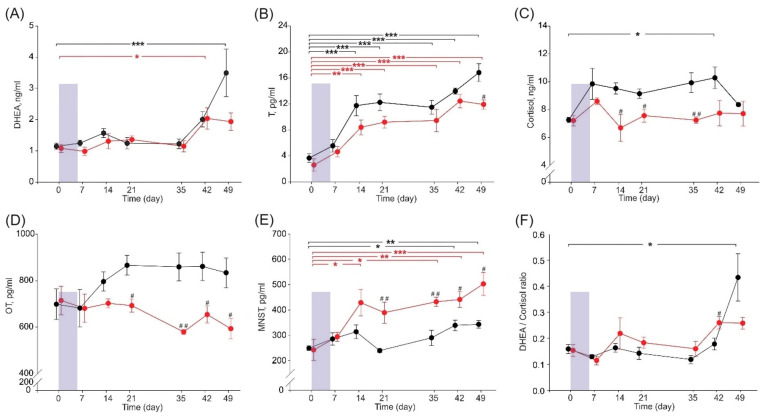
Time dependence of serum sarcopenic indicators during the experimental period: (**A**) dehydroepiandrosterone, DHEA; (**B**) testosterone, T; (**C**) cortisol; (**D**) oxytocin, OT; (**E**) myostatin, MNST; and (**F**) DHEA/cortisol ratio in pigs during the experimental period. Data are shown for the control group (C-pigs, black) and the cerulein-injected group (SSARC-pigs, red). The shaded area represents the 6-day-long period of daily cerulein injections (1 µg/kg b.w./day) in the SSARC-pig group. Data are presented as mean ± SE (n = 5 in each group). Statistical significance: * *p* < 0.05; ** *p* < 0.01; *** *p* < 0.001 (compared to day 0); # *p* < 0.05; ## *p* < 0.01 (between groups at a given time point).

Serum DHEA concentration increased over time and peaked on day 42 and day 49 in SSARC-pigs and C-pigs, respectively, compared to that observed on day 0 (Figure 2A). The serum T concentration increased gradually over time in both pig groups, with increased concentrations detected on days 14, 21, 35, 42, and 49 compared to that noted on day 0. Moreover, the serum T concentration in SSARC-pigs on day 49 was significantly lower compared to that of C-pigs (Figure 2B). The serum cortisol concentration was significantly increased in C-pigs on day 42 compared to day 0. Moreover, serum cortisol concentration was significantly decreased on days 14, 21, and 35 in SSARC-pigs compared to C-pigs on the same days (Figure 2C). Serum OT concentration was significantly lower in SSARC-pigs on days 21, 35, 42, and 49 compared to that observed in C-pigs on the same days (Figure 2D). SSARC-pigs had significantly increased serum MNST concentrations on days 14, 35, 42, and 49 compared to that observed on day 0. Serum MNST concentration was also increased in C-pigs on day 42 and day 49 compared to that noted on day 0. Moreover, the serum MNST concentration was significantly higher in SSARC-pigs on days 21, 35, 42, and 49 compared to that observed in C-pigs on the same days (Figure 2E). The DHEA/cortisol ratio was increased in C-pigs on day 49 compared to the ratio calculated on day 0, and this ratio was significantly higher in SSARC-pigs on day 42 compared to that of the C-pigs at this time (Figure 2F).

### 2.3. Expression of Genes of Antioxidant Proteins and Signal Proteins of Programmed Cell Death

**Figure 3:**
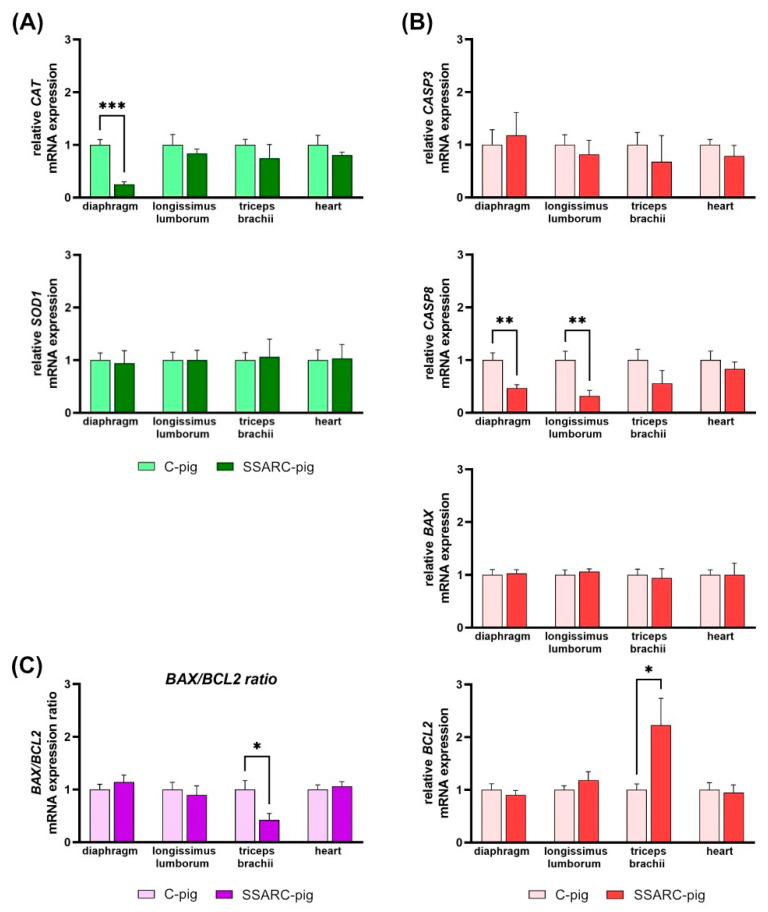
Relative gene expression of (**A**) antioxidant proteins, (**B**) signal proteins of programmed cell death, and (**C**) BAX/BCL2 mRNA ratio in pigs in the control (C-pig) and cerulein-injected (SSARC-pig) groups. The expression was normalized to the GAPDH housekeeping gene and is presented relative to the level observed in the control group. Due to the −ΔΔCT method’s exponential nature for calculating relative mRNA expression, the graphs depict the geometric means with SE (standard errors) (n = 5 in each group). Statistical significance: * *p* < 0.05, ** *p* < 0.01, *** *p* < 0.001 (two-tailed *t*-test adjusted for multiple comparisons using the Bonferroni method).

In the examined mRNA gene expression of antioxidant proteins, CAT mRNA was downregulated in the diaphragm of SSARC-pigs (Figure 3A). For cell death signaling proteins, CASP8 mRNA was downregulated in the diaphragm and ML of SSARC-pigs. Conversely, BCL-2 mRNA was upregulated in the MT of SSARC-pigs (Figure 3B). Consequently, the BAX/BCL-2 mRNA ratio in MT decreased for SSARC-pigs (Figure 3C).

## 3. Discussion

The application of CT or MRI imaging methods in research involving young pigs is complicated by issues related to population heterogeneity and varying research conditions. There is still a significant gap in validated methods for assessing muscle functionality because portable, hand-held dynamometers and manual muscle testers, often used in human studies, lack validation for use in porcine models. Similarly, in vivo techniques, such as electrical stimulation in anesthetized subjects, which offer some insight into muscle strength, are not yet fully validated for use in large animal models. Therefore, this study focused on assessing structural and molecular changes in the muscles. This comprehensive approach allowed for a detailed investigation into how CP affects muscle structure and function through oxidative stress and programmed cell death mechanisms.

As mentioned, pancreatitis is a systemic inflammation and one of the most common problems in gastroenterology due to its often asymptomatic onset [37,38,39,40]. Various data indicate that its prevalence in children is approximately 5.8 per 100,000 among privately insured children in the USA [41,42], while the prevalence of sarcopenia in CP from all studies ranged from 17–62% [38], and it is more frequently studied in older people than in youths. Despite its clinical significance, research on youth-onset sarcopenia remains in its infancy [43]. Tracking changes in the musculoskeletal system in humans is difficult, as is obtaining diaphragm or heart samples by biopsy. Experiments on animals allow for the discovery of previously unknown mechanisms or effects [44,45]. The choice of the pig as the experimental animal is justified because pig physiology is very similar to that of humans, and pigs are close in body size to humans [46,47]. Moreover, pigs are more developed at birth than rodents; the number and size of muscle fibers at birth are fixed [48]. Pigs share a similar developmental pattern of skeletal muscles, making them advantageous models for studying pathophysiological conditions in humans [49,50,51]. All of these conditions favor the selection of the pig to carry out the present study. On the other hand, the young age of the animals means they are still in a critical phase of growth and development. This developmental stage might influence muscle fiber composition and response to stress differently than in fully mature animals. Natural growth processes could mask the extent of the sarcopenic changes observed.

In general, skeletal muscles account for about 55% of the body mass in mammals. They play a major role in locomotion, protect internal vital organs, provide body posture and shape (also related to sex), participate in heat production and thermoregulation, respond to cold stress, and are involved in bodily metabolism [52]. Skeletal muscles, based on their contractile and metabolic properties, are roughly classified as slow-twitch oxidative (type Ⅰ) fibers, fast-twitch oxidative–glycolytic fibers (type Ⅱa), and fast-twitch glycolytic fibers (type Ⅱb) in pigs. In humans, however, type IIx replaces type IIb as the dominant fast-twitch fiber (with IIx previously called IIb) [50,53,54,55]. Type I fibers are characterized by high endurance, slow contraction speed, high oxidative capacity, numerous mitochondria, and high myoglobin content. They are resistant to fatigue and predominantly used for prolonged, low-intensity activities and maintaining posture. Type IIa fibers exhibit intermediate endurance, fast contraction speed and oxidative and glycolytic metabolism; they are relatively resistant to fatigue, making them suitable for sustained activities and short bursts of moderate-intensity exercise. Type IIb fibers, on the other hand, have low endurance, very fast contraction speed, and high glycolytic capacity; they are prone to fatigue, being utilized for short, high-intensity activities [50]. The proportion between all these fibers can vary among different species, and in humans, this proportion shows variability between individuals. Additionally, the anatomical position of muscles determines their fiber pattern and role in physical activity (e.g., trunk, limb, head, or neck muscles) [50,56]. It is also known that the type of skeletal muscle fiber shifts in response to different health problems or immobility in humans [52]. In livestock animals, fiber composition is a major determinant of meat quality [57].

The current study focused on muscles consisting of three types of fibers: LL [58], contributing to lumbar postural control both in pigs and humans [59]; TB [60], involved in studies relating to strength training, training adaptation, and aging [61] due to its more dynamic function in physical activity [62,63]; the diaphragm, which responds to health problems by reducing its diameter [64,65]; and the heart, characterized by fibers with different contractile and conducting properties [66]. The current study showed an increase in the number of fibers in LL and a decrease in the diameter of both type II fibers in LL, all types in TB, and type I and IIa in the diaphragm, without a change in the percentage composition of muscle fibers. The fiber types affected in all muscles analyzed were type IIa, a fast-twitch type with high oxidative and glycolytic capacity and relatively resistant to fatigue. This type produces ATP through oxidative phosphorylation and anaerobic glycolysis, which is essential for sustaining prolonged physical activity and endurance exercises and allows it to perform well during high-intensity, short-duration activities, where oxygen availability might be limited. The reduction in the number and diameter of type IIa fibers means that muscles may generate less force, leading to an overall weakening of muscle strength and faster fatigue during physical exertion. The combination of reduced strength and endurance can lead to mobility issues and decrease quality of life, affecting the ability to perform daily tasks and activities, which is especially problematic for individuals with chronic diseases. Moreover, the total number of fibers in a given muscle area may increase. This can be explained by the reduction in the diameter of individual muscle fibers resulting from degeneration and decreased protein synthesis in the muscles. The reduced thickness of the fibers means that more fibers can fit into the same area. It should be noted that, even though the number of fibers per unit area increases, the overall muscle strength and its ability to generate power are still reduced.

This result is consistent with other data showing a reduction in size, particularly in type II fibers, which are the predominant type, especially IIb, in domestic pigs [67]. Additionally, PSARC is linked with the decrease in type II fibers, explained by the shift from type II to type I, mainly observed with age [13]. There is also a report showing that type I fibers, predominant in postural muscles, are more susceptible to sarcopenic changes [68]. However, the pattern of muscle atrophy differs among studies and individuals [69,70,71]. The latest findings show that SARC affects the skeletal muscles of the lower limbs more than the predominantly type II fibers in the upper part of the body in humans [72,73]. Moreover, taking into account that CP is associated with malabsorption and nutrient deficiencies due to impaired digestive enzyme secretion and altered intestinal permeability, this reduced nutrient availability can significantly affect muscle metabolism, regeneration, and overall health. The balance between muscle protein synthesis and breakdown determines muscle mass [11]. Insufficient nutrients may exacerbate sarcopenic changes, as muscles require adequate protein and other nutrients to maintain mass and function [74]. The significant difference in body weight between the experimental and control groups at an age of very intense muscle mass growth could be due to both inhibited muscle mass growth and its reduction. However, our growing pigs may have experienced sarcopenic changes rather than cachexia or growth inhibition because a previous study showed no pain, diarrhea, or decreased food intake, and similar serum biochemical indices (protein and albumin levels), indicating comparable nutritional status [35]. While microscopic analysis indicated a decrease in fiber diameter without changes in composition, the duration may have been too short for these changes to fully manifest because SSARC is categorized into three severity stages, according to the European Working Group on Sarcopenia. The current findings were typical for the pre-sarcopenia stage comprising muscle mass reduction alone [75].

Tumor necrosis factor alpha, considered a potent inducer of muscle wasting in vivo through the induction of apoptosis and proteolysis and the inhibition of myogenesis [76], increases in patients with CP [77]. Interleukin-6 is a catabolic agent involved in muscle wasting and, inhibited by DHEA, it increases with age [78,79,80]. Interleukin-1beta is also elevated in CP, acting as a mediator of systemic inflammation [81]. All of these factors are some of the most important biomarkers of sarcopenia [82]. In addition to CRP, LDH, GGTP, SOD, and GSH, they all increased after cerulein injections, as reported previously [35]. However, considering the complexity of SARC, it is unlikely that a single biomarker can identify the sarcopenic condition in a heterogeneous population of young and old individuals [83]. Different hormones affect muscle function and mass. Many hormones, such as sex hormones, which are muscle-growth promotors and regulators of regenerative factors, along with T [84] and its precursor DHEA, [85] decline in sarcopenia [86]. Sex steroid hormones may participate in intracellular signaling pathways, such as the IGF-1/Akt/mTOR pathway, MAPK pathway, and Wnt and Notch signaling, which either positively or negatively regulate cell proliferation, survival, and energy metabolism [87]. DHEA mitigates oxidative stress and increased GSH [88]. In the current study, SSARC-pigs showed distinct hormonal profiles with significant alterations in DHEA, T, cortisol, OT, and MNST levels over time compared to controls. In addition to increased proinflammatory factors, this may be another reason for the reduced diameter of the muscle fibers.

The roles of cortisol and dysfunction of the HPA axis in the pathogenesis of SARC in aging individuals are well established [89,90]. Cortisol is a well-known catabolic hormone that induces muscle atrophy and wasting [86]. Both 11beta-hydroxysteroid dehydrogenase (which interconverts the active glucocorticoid, cortisol, and inactive cortisone) and 5alpha-reductase (which plays a key role in cortisol breakdown and reduces testosterone to dihydrotestosterone) are involved in cortisol metabolism, and both decline in aging men [91]. However, the current study involved young pigs, in which the cortisol concentration remained relatively constant through the whole study. It should be emphasized that the study was conducted in such a way as to eliminate as many stress factors as possible. Although the DHEA/cortisol ratio changed in a comparable manner in both groups, cortisol was lower and the DHEA/cortisol ratio was significantly higher in SSARC-pigs on day 42. Future studies should be prolonged to clarify serum cortisol changes, especially since individual responses to stress may vary [92]. On the other hand, it is not known whether a DHEA-S/cortisol value higher than 0.2, an independent risk factor for SARC, can be extrapolated to pigs [93]. However, there is a basic mechanism in which high cortisol induces catabolic effects, while low DHEA reduces anabolic reactions in skeletal muscles [93].

Further, changes in OT and MNST were assessed over time in the current study. The observed hormonal changes, such as decreased testosterone and OT levels and increased myostatin levels in SSARC-pigs, are critical indicators of muscle anabolic-catabolic balance. Testosterone and OT promote muscle growth and repair, while myostatin inhibits these processes. The hormonal imbalance observed in SSARC-pigs indicates a shift towards catabolism, contributing to sarcopenia. OT and MNST are sarcopenic indicators, and the obtained results are consistent with other data [94,95,96,97,98]. OT, a neuropeptide produced in magnocellular neurons of the supraoptic and paraventricular nuclei and additionally in VON in pigs, is released by the posterior pituitary [99]. It exerts a protective effect against ischemia or injury in skeletal muscles in addition to its role in reproductive physiology and social relations [100]. It is important for regeneration and the maintenance of muscle homeostasis. Genetic deficiency of OT has been linked with premature sarcopenia [97]. The myokine MNST is present not only in skeletal muscles but also in the heart [101]. MNST concentration does not reflect its activity because it is released in precursor form; the signal peptide must be removed to form the mature, biologically active form [102]. MNST is a key regulatory protein of muscle growth; when it is inhibited, muscle hypertrophy occurs. Its serum level is inversely related to skeletal muscle mass, although it is not considered a primary driver of SARC [102,103,104]. The increased MNST observed in SSARC-pigs, alongside the decreased levels of testosterone and OT, suggests a comprehensive shift towards muscle catabolism, indicating a synergistic effect leading to sarcopenia. The reduction in muscle fiber cross-sectional area observed in SSARC-pigs aligns with the increased MNST, which inhibits muscle growth. This provides a clear observation of how the altered hormonal state related to muscle growth and repair processes manifests in the muscle structure.

As mentioned above, the diaphragm is very sensitive to various health problems. The current study showed that the mRNA expression of the *CAT* gene decreased in the diaphragm. This suggests a decreased synthesis of catalase. The decrease in *CAT* mRNA in the diaphragm suggests impaired antioxidant defense, which can lead to increased oxidative damage characteristic of sarcopenia [105,106]. Oxidative stress can activate an apoptotic signaling pathway linking CP to sarcopenia in muscle cells through several mechanisms. Through the intrinsic (mitochondrial) pathway, oxidative stress damages mitochondria, causing the release of cytochrome c and the activation of caspase-9. This activation initiates a caspase cascade, including caspase-3, leading to apoptosis and subsequent muscle cell death. The extrinsic (death receptor) pathway involves proinflammatory cytokines, such as TNF-α, binding to death receptors (e.g., TNF receptor 1) on muscle cells. This binding activates caspase-8, which in turn activates downstream caspases, resulting in apoptosis. Moreover, oxidative stress can cause endoplasmic reticulum (ER) stress, leading to the activation of the unfolded protein response [107]. Prolonged ER stress activates apoptotic pathways through caspase-12, contributing to muscle cell death. Thus, the combined effects of oxidative stress and apoptosis result in muscle degradation and loss of muscle mass and function, characteristic of sarcopenia. Persistent oxidative stress and apoptosis lead to a reduction in muscle fiber size and number, contributing to muscle atrophy. This atrophy is further exacerbated by reduced protein synthesis and increased proteolysis [108]. Muscle stem cells (satellite cells) are also affected by oxidative stress and apoptosis, impairing their ability to regenerate damaged muscle tissue. This results in a diminished capacity for muscle repair and regeneration. Consequently, the losses of muscle mass and structural integrity of muscle fibers lead to decreased muscle strength and function, hallmarks of sarcopenia [109].

The death of individual nuclei in multi-nucleated skeletal muscles occurs without the elimination of the entire cell [110]. Apoptotic signaling in age-related sarcopenia mainly occurs through intrinsic and mitochondrial apoptotic pathways [111]. Myofibers and satellite cells are also susceptible to extrinsic nuclear apoptosis initiated by TNF-α and caspase-3 and act independently of mitochondrial signaling [110]. Skeletal muscle atrophy and the myofiber loss observed in age-related sarcopenia result from apoptosis involved in the loss of muscle fiber nuclei, initiated by caspace-3, which is affected by caspase-8 and TNF-α [111,112], which increased in SARC. The increase in proinflammatory factors in SARC has led to the term “inflammaging” [113]. Additionally, pyroptosis-mediated inflammation, as a form of programmed cell death, is considered [114,115,116]. Pyroptosis-mediated inflammation is driven by IL-18 and IL-1β, which are overproduced by the activation of caspase-1 [114]. Caspases involving pathway signaling are well-described in age-related SARC. In young individuals, the intracellular mechanisms of atrophy rely on caspase-independent pathways [111]. The lack of changes in the mRNA expression of the *CASP3* gene and reduction in the mRNA expression of the *CASP8* gene in SSARC-pigs could support this finding. The downregulation of *CASP8* in the diaphragm and ML indicates reduced apoptotic signaling, which may reflect a compensatory mechanism to prevent excessive cell death under chronic stress. To determine whether the sarcopenic effect in muscles in young pigs was linked to caspase-independent apoptotic signaling or pyroptosis-mediated inflammation, further investigation of caspase-1 and IL-18 is needed.

Apoptosis is regulated by an expanding family of BCL-2 proteins, which include both proapoptotic and antiapoptotic members and act as checkpoints upstream of caspases and mitochondrial dysfunction [117]. Among these, Bcl-2 is an antiapoptotic protein known for its prosurvival activity [118]. Bcl-2 is localized to mitochondria, the endoplasmic reticulum, and nuclear membranes, which are also the sites of reactive oxygen species generation. Bcl-2 does not affect the generation of oxygen free radicals, but helps prevent oxidative damage [119]. Studies have shown a reduction of Bcl-2 levels in aged rats, contrary to that in young exercising rats [120]. Conversely, the process of caspase-independent programmed cell death (PCD) is regulated by Bcl-2 family proteins [121]. Mitochondria, as central integrators of PCD signaling pathways, release various factors that trigger caspase-independent cell death. PCD can also be induced by altered mitochondrial energetics, such as the loss of cytochrome c [121]. Bax is a proapoptotic family Bcl-2 protein whose expression increases in response to apoptotic stimuli [122]. It is important to note that there is a network of interactions between apoptosis and autophagy pathways in which the Bcl-2 protein family plays an important role. In addition to their proapoptotic and antiapoptotic functions, Bcl-2 family proteins also induce or inhibit autophagy through their interactions with Beclin 1.

The results showed an increase in the expression of the antiapoptotic *BCL2* gene, while the expression of the proapoptotic *BAX* gene remained unchanged in the MT of young SSARC-pigs, where all fibers were affected. The *BAX*/*BCL2* ratio suggests that the signaling pathways in SSARC-pig could be caspase-independent, considering the role of this ratio in regulating caspase-3. The upregulation of *BCL2* in MT indicates an antiapoptotic response, potentially aimed at protecting muscle integrity. However, beyond their proapoptotic and antiapoptotic functions, the Bcl-2 protein family could also inhibit autophagy indirectly by inhibiting Bax. Given the young age of animals, further investigation is needed.

The combined analysis of hormonal changes, gene expression profiles, and muscle fiber characteristics offers a comprehensive understanding of sarcopenia in SSARC-pigs. The hormonal shifts toward catabolism, along with changes in gene expression and muscle fiber atrophy, reveal the multifaceted nature of muscle degradation in chronic illness. Integrating these findings provides a more holistic view of the mechanisms underlying SARC in the context of chronic illness. However, these factors must be carefully considered to accurately interpret the extent and implications of sarcopenic changes in this model. Nutrient deficiencies could impair muscle protein synthesis and repair mechanisms, contributing to muscle atrophy and weakness. The observed changes in muscle fiber diameters and gene expression related to oxidative stress and apoptosis might be more pronounced due to these nutritional challenges, similar to the problems observed in chronic diseases in malnourished adults.

Although the study has some limitations, including 1. the lack of imaging methods, such as CT or MRI, which are considered the gold standard in human medicine, 2. the challenges associated with assessing muscle strength functionally in animals, and 3. the absence of techniques, such as ELISA or Western Blot, to confirm changes in selected mRNA expression related to programmed cell death and antioxidative markers, it also has several strengths, such as 1. histological analysis of muscle fibers, 2. immunohistochemical differentiation between muscle fiber types, 3. (most importantly) the evaluation of various muscle types, and 4. the integration of hormonal, molecular, and structural data, which provides a comprehensive perspective on muscle degradation processes. However, future research should address this study’s limitations to enhance the validity and applicability of the findings.

The current study demonstrated that CP in young growing pigs resulted in the reduction of various muscle fibers, with variations in the pattern of muscle fiber reduction observed among different muscles. Gene expression of the proteins involved in PCD varied among different muscles, indicating that further research is recommended to determine whether the effects of SARC in the muscles of young pigs with CP are related to a caspase-independent apoptotic signal or to inflammation mediated by pyroptosis.

## 4. Materials and Methods

### 4.1. Animals and Treatment Groups

The study involved 10 healthy, uncastrated boars (Polish Landrace, pbz), aged 9–10 weeks. Following a 7-day acclimation period, the pigs were divided into two groups: the control group (the C-pig group), and those subjected to chronic pancreatic (CP) induction (the SSARC-pig group). Control pigs, matched for age, sex, and weight, served as the basis for comparison against the CP-inducted inflammatory changes noted in SSARC-pigs. SSARC-pigs received intramuscular cerulein injections (Caerulein, C2389, Sigma-Aldrich Merck KGaA, Darmstadt, Germany), dissolved in vehicle (saline solution), at a dosage of 1 µg/kg b.w./day for six consecutive days, with 24-h intervals between doses [123]. In contrast, the control pigs received only the vehicle.

After the injection phase, the pigs from both groups were maintained for the next 6 weeks. Both animal groups received identical feed intended for pigs of this age, encompassing all essential nutrients as recommended by the NRC and Polish nutrition standards for pigs [124,125]. Unrestricted access to water was provided through nipple drinkers. Blood samples were collected weekly, a total of five times, excluding day 28 (week 4), the third week after stopping the cerulein injections, due to COVID-19 restrictions. Given diurnal variations in cortisol, blood was consistently drawn in the morning to minimize inconsistencies. The coagulated blood was centrifuged (1300× *g* for 10 min at 18 °C) to obtain serum, which was aliquoted and stored at −86 °C.

At the end of the experiment (day 49, or 43 days post-cerulein administration), all animals were subjected to pharmacological euthanasia via i.m. injections of ketamine (Biowet, Puławy, Poland) at a dose of 350 mg/100 kg b.w., xylazine (Sedazin, Biowet, Puławy, Poland), at a dose of 200 mg/100 kg b.w.), azaperone (Stresnil, Elanco GmbH, Cuxhaven, Germany) at a dose of 30 mg/100 kg b.w., and an i.v. dose of pentobarbital (Morbital 26.7 mg/mL; Biowet, Puławy, Poland) at 0.3–0.6 mL/kg b.w.

### 4.2. Serum Parameters

The blood serum concentrations of dehydroepiandrosterone (DHEA), testosterone (T), oxytocin (OT), myostatin (MNST), and cortisol were quantified. Pig-specific enzyme-linked immunosorbent assay (ELISA) kits were used. For DHEA (#EU2945, intra-assay CV < 8%, inter-assay CV < 10%), T (#EU0400, intra-assay CV < 8%, inter-assay CV < 10%), OT (#EU2549), and MNST (#EP0354), kits were sourced from Wuhan Fine Biotech Co., Ltd., Wuhan, China. The cortisol kit was obtained from Qayee-bio, Shanghai, China (#QY-E40032). All assays were performed in three technical replicates following the manufacturer’s instructions rigorously. The intra- and interassay coefficients of variation (CVs) for all kits were below 8% and 10%, respectively. Absorbance readings were taken using a Benchmark Plus microplate spectrophotometer (Bio-Rad Laboratories, Inc., Hercules, CA, USA). The DHEA/cortisol ratio was determined to assess potential disruptions in the hypothalamic–pituitary–adrenal (HPA) axis due to chronic stress.

### 4.3. Muscle Sample Collection

Within 5 min of euthanasia of the pigs, muscle samples designated for microstructural and gene mRNA expression analysis were harvested from the right side of the carcass. From the longissimus lumborum muscle (ML), samples were obtained at the first lumbar vertebra level, specifically from the muscle’s central portion. Similarly, samples from the central section of the triceps brachii muscle (caput longum) (MT), the heart’s lateral wall, and the right crus of the diaphragm were collected. For histochemical and immunohistochemical studies, muscle specimens were sectioned into 1 cm^3^ fragments, parallel to the direction of the muscle fibers. They were then flash-frozen in isopentane (Sigma-Aldrich Merck KGaA, Darmstadt, Germany), pre-chilled using liquid nitrogen, and stored at −86 °C for subsequent analysis. The muscle specimens designated for mRNA analysis were promptly submerged in StayRNA (#038, A&A Biotechnology, Gdynia, Poland) and preserved at −20 °C until the isolation of total RNA.

### 4.4. Muscle Histochemical and Immunohistochemical Analyses

Muscle samples were fixed to a cryostat chuck using a few droplets of tissue freezing medium (Leica Biosystems GmbH, Nussloch, Germany), and then sectioned at −20 °C in a cryostat (Slee MEV, Mainz, Germany) into 10-μm thick transverse slices. These sections were subsequently placed on microscope slides. To differentiate muscle fiber types, a modified combined technique encompassing both nicotinamide adenine dinucleotide tetrazolium reductase (NADH-TR) activity and immunohistochemical (IHC) identification of the slow myosin heavy chain on the same section was utilized [126]. Initially, for NADH-TR determination, the sections were allowed to air-dry for 1 h at 37 °C. Subsequently, they were incubated for 1 h at 37 °C in a medium consisting of 6 mg NADH (β-Nicotinamide adenine dinucleotide, N8129, Sigma-Aldrich, St. Louis, MO, USA), 0.25 mL NBT (nitro-blue tetrazolium, N6876, Sigma-Aldrich, St. Louis, MO, USA) dissolved in ddH_2_O to achieve a concentration of 1 mg/mL, 0.25 mL of 0.2 M Tris buffer (pH 8.0), and 0.5 mL ddH_2_O [127]. After the incubation period, the sections were washed with three exchanges of ddH_2_O. This reaction identifies three fiber types: I, IIa, and IIb. Type I fibers exhibit a darker blue shade compared to type IIb (which remains unstained), while type IIa fibers present a medium blue intensity. To ensure accurate differentiation between type I and type IIa fibers, the same sections underwent an additional IHC reaction. This employed a mouse monoclonal antibody specific to the myosin heavy chain slow/I isoforms (NCL-MHCs, Leica Biosystems GmbH, Nussloch, Germany; diluted 1:80 in Tris buffer, pH 7.6). The preparations were first incubated with primary antibody for 1 h in RT. Visualization was achieved using the NovoLink Polymer Detection System (Leica Biosystems GmbH, Nussloch, Germany), as per the manufacturer’s instructions. The IHC reaction differentiated type I fibers (showing a positive IHC reaction for slow myosin) from type IIa and IIb fibers (no reaction). Finally, all slices were dehydrated through a graded series of EtOH (75%, 96%, and 100%), cleared in xylene, and set in a mounting medium (DPX, Sigma-Aldrich, St. Louis, MO, USA). Through this combined methodology, the three distinct muscle fiber types were discernible: brown-blue granulated (type I), blue granulated (type IIa), and unstained (type IIb).

For the measurement of the diameters of different muscle fiber types, at least 300 fibers from a minimum of 10 muscle bundles were analyzed per section. The proportion of each muscle fiber type was calculated by dividing the count of fibers of a specific type by the total number of muscle fibers present in each bundle. The number of muscle fibers was calculated per mm^2^ of the muscle transverse cross-section. In each section, 10 randomly selected areas were evaluated, and the mean number per individual was calculated. All muscle fiber characteristics were examined using a light microscope (Nikon E600, Nikon, Tokyo, Japan) and quantified with MultiScan image analysis software (v. 14.02, Computer Scanning Systems Ltd., Warsaw, Poland). The person performing the measurements was blinded to the specific treatment.

### 4.5. RNA Isolation and mRNA Quantification

Total RNA was extracted according to the Chomczynski and Sacchi method [128]. The muscle samples were homogenized in Eppendorf tubes using an Ultra Turrax T25 dispenser (IKA Labortechnik, Staufen im Breisgau, Germany) in TRI Reagent (T9424, Sigma-Aldrich Merck KGaA, Darmstadt, Germany) using a ratio of 1 mL TRI per 100 mg of tissue. To achieve phase separation, 50 μL of BCP (1-Bromo-3-chloropropane, B9673, Sigma-Aldrich, St. Louis, MO, USA) was added. After vigorous shaking, the tubes were placed on ice for 15 min. Subsequent centrifugation was performed (5425R Eppendorf, Hamburg, Germany) at 12,000× *g* for 15 min at 4 °C. The resulting aqueous phase containing RNA was transferred to sterile tubes. For RNA precipitation, 0.5 mL of isopropyl alcohol was added, and the samples were refrigerated overnight. The following day, they were centrifuged again (12,000× *g* for 15 min at 4 °C). After careful removal of the isopropyl alcohol, the RNA was washed with 75% EtOH and centrifuged (7600× *g* for 8 min at 4 °C). The ETOH was discarded, and the RNA was allowed to dry in open tubes on ice for approximately 5 min. The RNA was then dissolved in RNAse- and DNAse-free PCR-grade water and incubated at 55 °C for around 12 min. The integrity of the total RNA was assessed using 2% agarose gel electrophoresis. The quality and concentration of the extracted RNA were ascertained using spectrophotometric analysis (Nanodrop Lite Spectrophotometer, Thermo Fisher Scientific, Waltham, MA, USA) at wavelengths of 260 and 280 nm. All sample absorbance ratios for 260/280 were in the range of 1.9–2.0.

Reverse transcription was conducted as previously described [129]. Briefly, 2 µg of total RNA was reverse-transcribed using the High-Capacity cDNA Reverse Transcription Kit (4368814, ThermoFisher Scientific, Waltham, MA, USA) with random primers. The reaction conditions in a thermocycler (Mastercycler Gradient; Eppendorf, Hamburg, Germany) were set at 25 °C for 10 min, 37 °C for 120 min, and 85 °C for 5 min. The obtained cDNA was used in real-time qPCR to assess the expression of superoxide dismutase [Cu-Zn] (*SOD1*), catalase (*CAT*), caspase-3 (*CASP3*), caspase-8 (*CASP8*), Bcl-2 (*BLC2*), and Bax (*BAX*) with glyceraldeyde-3-phosphate dehydrogenase (*GAPDH*) serving as the housekeeping gene. Primers used for amplification (Table 1) were synthesized by IBB PAS (Institute of Biochemistry and Biophysics of the Polish Academy of Sciences, Warsaw, Poland) according to previously validated sequences [130,131]. The qPCR reactions for the targeted genes were set up in 10 µL volumes that comprised 1 µL of 10× diluted cDNA, 2 µL of 5× HOT FIREPol EvaGreen qPCR Mix Plus (08-24-00001, Solis BioDyne, Tartu, Estonia), 0.12 µL each of forward and reverse primers (10 pmol/μL), and the volume was made up to 10 µL with PCR grade water. Reactions for each of the samples were carried out with three technical replicates. A no-template control was included in each run to verify the absence of sample/reagents contamination.

The relative expression level of the examined genes in muscles was calculated after normalization with the GAPDH transcript using the −ΔΔCT method [132]. The analysis was performed using the integrated software from StepOne (v2.3., Applied Biosystems, Walthman, MA, USA). The *BAX*/*BCL2* mRNA ratio was also calculated as an indicator of apoptosis [133,134].

### 4.6. Statistical Analysis

Each individual animal was considered an experimental unit for all tests (n = 5 in each group). Preliminary tests for data normality and variance homogeneity were conducted using the Shapiro–Wilk and Levene’s tests, respectively. For serum parameters, variations over time were analyzed using a repeated measures ANOVA, with blood sampling time as the main factor. Differences between initial values (taken at week 0) and subsequent data points within the same group were determined using Tukey’s HSD post hoc test. The effect of CP induction was assessed by contrasting the C-pig group with the SSARC-pig group, using a one-way ANOVA followed by Tukey’s HSD post hoc test. Considering the multiple observations for muscle fiber data within a single individual, a mixed-model ANOVA was adopted. In this model, the experimental group (C-pig or SSARC-pig) was treated as a fixed effect, while the animal was treated as a random effect. This linear mixed-effects model ensured that each pig (n = 5 in each group) was considered the experimental unit in the ANOVA. When significantly different, a post hoc Tukey’s HSD adjustment was used to compare the means. Differences in gene expression within specific muscle types between groups were assessed using an unpaired two-tailed Student’s *t*-test, adjusted for multiple tests using the Bonferroni correction.

The statistical analyses were conducted using Statistica software (ver. 13.0, Tibco Software Inc., Palo Alto, CA, USA). A significance level of *p* < 0.05 was adopted for all tests. For graphical representations of the data, GraphPad Prism (ver. 9.5.1, GraphPad Software, San Diego, CA, USA) was utilized. All values are presented as the mean ± SEM.

## 5. Conclusions

The study underscores that CP induction leads to a reduction in muscle fibers in LL and a decrease in the diameter of type IIa muscle fibers across various muscles, indicating sarcopenic processes. This reduction in fiber number suggests a decline in muscle strength and endurance. Additionally, significant alterations in gene expression patterns related to apoptosis and oxidative stress were identified along with hormonal shifts favoring catabolism. These molecular and hormonal changes contribute to muscle degeneration. Elevated levels of proinflammatory cytokines and decreased antioxidant levels further highlight the crucial role of inflammation and oxidative stress in the development of sarcopenia associated with CP.

## Figures and Tables

**Table 1:** Primers used in this study.

Gene	Primer Sequences (5′ to 3′)	Product Size (bp)	GeneBank Accession Number	Reference
*SOD1*	F: CGAGCTGAAGGGAGAGAAGA R: ACATTGCCCAGGTCTCCAA	199	NM_0 01190422.1	[130]
*CAT*	F: ATGTGCAGGCTGGATCTCAC R: GCACAGGAGAATCTTGCATC	155	XM_021081498.1	[130]
*CASP3*	F: CAAGTTTCTTCAGAGGGGACTGC R: TCGCCAGGAATAGTAACCAGGTGC	202	NM_214131	[131]
*CASP8*	F: TCCCAGGATTTGCCTC R: AAGCCAGGTCATCACTGTC	112	NM_001031779	[131]
*BCL2*	F: AGGGCATTCAGTGACCTGAC R: CGATCCGACTCACCAATAC	193	NM_214285	[130]
*BAX*	F: TGCCTCAGGATGCATCTACC R: AAGTAGAAAAGCGCGACCA	199	XM_003127290	[130]
*GAPDH*	F: ATCCCGCCAACATCAAAT R: TCACGCCCATCACAAACA	165	XM_021091114.1	[130]

*SOD1*—superoxide dismutase [Cu-Zn], *CAT*—catalase, *CASP3*—caspase-3, *CASP8*—caspase-8, *BCL2*—B-cell lymphoma 2, *BAX*—BCL2 associated X, apoptosis regulator, *GAPDH*—glyceraldehyde 3-phosphate dehydrogenase (housekeeping gene).

## Data Availability

The data presented in this study are available on request from the corresponding author.
